# Influences of Cheek Support on Sucking Pressure During Bottle Feeding in Infants: A Pilot Study

**DOI:** 10.24546/0100499756

**Published:** 2026-02-03

**Authors:** TAKESHI KOMATSU, TATSUYA FURUKAWA, DAISUKE KOBAYASHI, SOTA IWATANI, SEIJI YOSHIMOTO, TOSHIHIKO YAMASHITA, SAYAKA KATSUNUMA, MASAHIDE OTSU, TAKESHI FUJITA, KEN-ICHI NIBU

**Affiliations:** 1 Department of Rehabilitation, Hyogo Prefectural Kakogawa Medical Center, Kakogawa, Japan; 2 Department of Otolaryngology-Head and Neck Surgery, Kobe University Graduate School of Medicine, Kobe, Japan; 3 Department of Pediatric Rehabilitation and Orthopedics, Hyogo Prefectural Kobe Children’s Hospital, Kobe, Japan; 4 Department of Neonatology, Hyogo Prefectural Kobe Children’s Hospital, Kobe, Japan; 5 Department of Otolaryngology, Hyogo Prefectural Kobe Children’s Hospital, Kobe, Japan

**Keywords:** Sucking pressure, Cheek support, Oral support, Feeding, Infant, Swallowing function

## Abstract

Effective sucking is important for shortening hospital stays in newborn infants. Several studies have demonstrated that oral support can improve sucking efficiency. However, the effects of oral support on sucking pressure have not been thoroughly investigated. In our previous study, we reported the equipment configuration and analysis method of sucking pressure. That method of measuring sucking pressure was applied to cheek support. In this study, four newborn infants with low birth weight, congenital diaphragmatic hernia, or multicystic encephalomalacia were enrolled. Their sucking pressures were measured during bottle feeding, both with and without cheek support. In all cases, cheek support increased sucking pressure. However, changes in intake volume and the number of sucking bursts, individual sucks did not show a consistent pattern. Our study indicates a possible association between cheek support and sucking pressure during oral feeding in newborn infants. Larger studies are needed to confirm this effect.

## INTRODUCTION

Ensuring adequate oral nutrition is important for shortening hospital stays in newborn infants. Efficient and vigorous sucking, consisting of both compression and suction (1), is essential for successful oral feeding.

However, feeding difficulties may arise due to a variety of factors, such as prematurity (2), impaired respiration (3) and/or cardiorespiratory function (4), as well as gastrointestinal (5, 6), neurological (7, 8), or chromosomal abnormalities (9). Moreover, feeding difficulties can also occur in normally developing infants (1).

Several studies have demonstrated that oral support techniques, such as pressing cheeks (cheek support) and holding jaw (jaw support), are effective in enhancing sucking efficiency (1, 10–12). There were cautionary findings such as the decrease in SpO2 levels at the initiation of the feeding (12) and insertion of poses to facilitate breathing (1). Cheek support has been reported to improve suction by stabilizing the cheeks and promoting lip seal (13), whereas jaw support provides mandibular stability (1).

However, previous studies have primarily focused on preterm infants, and the methods of oral support used were inconsistent. Therefore, the individual effects of cheek and jaw support require further validation (11).

Additionally, although several studies (1, 14–17) have evaluated sucking pressure to assess infant oral feeding, none have investigated the effects of oral support on sucking pressure during feeding.

In this study, we report the effects of oral support, especially cheek support, on oral feeding by measuring sucking pressure in four infants with preterm birth, congenital diaphragmatic hernia, or multicystic encephalomalacia.

## MATERIALS AND METHODS

### Participants

We recruited newborn infants who were hospitalized in the NICU (Neonatal Intensive Care Unit) or GCU (Growing Care Unit) of the Comprehensive Perinatal Maternal and Child Medical Center at Kobe Children’s Hospital between April 2019 and November 2020. Among them, three were born with low birth weight. One infant had congenital diaphragmatic hernia, and another had multicystic encephalomalacia.

The enrolled infants had no congenital abnormalities of the oral cavity, nasal cavity, or larynx, and no comorbidities such as aspiration, apnea, bradycardia, or desaturation that could interfere with bottle feeding. Infants who had already transitioned from tube feeding and were able to feed by bottle were considered as candidates. Among them, only those who did not show obvious desaturation or bradycardia during routine feeding and who exhibited no abnormal muscle tone in the whole body or in the neck/oral region during everyday feeding were included in the study. Oral support was approved for all infants by their attending physicians.

The ethical committee of Hyogo Prefectural Kobe Children’s Hospital approved this study (approval code R2-10), and written informed consent was obtained from the parents of all the participants.

### Measurement of sucking pressure and intake amount during bottle feeding

**Figure 1:**
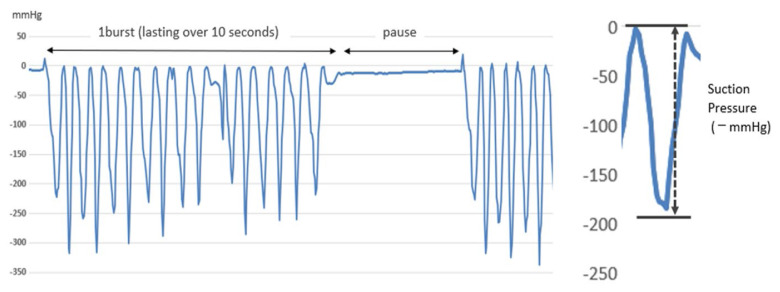
Pressure waveform for sucking pressure measurements Sucking Pressure: From the start of sucking to the bottom of the negative pressure waveform. Waveform: Starting from the pressure at the beginning of the sucking waveform and ending at the pressure at the end of the sucking waveform [mmHg × 0.05 s]

Sucking pressure was measured using a previously reported device developed by our team (17). An artificial nipple (SofTouch™ Peristaltic PLUS SSS size; Pigeon Co. Ltd., Tokyo, Japan), which is routinely used in nurseries, was used in this study. A silicone tube with an outer diameter of 2 mm and an inner diameter of 1 mm was attached 1 mm from the nipple hole at the tip of the artificial nipple, and a semiconductor pressure transducer PMS-5M-2™ (JTEKT Co. Ltd., Tokyo, Japan) was connected to the end of the tube. The pressures were amplified with an AA6210 amplifier (JTEKT Co. Ltd., Tokyo, Japan) and recorded using a data logger (GL240; Graphtec Co. Ltd., Yokohama, Japan). Measurements were recorded for 5 minutes immediately after the start of the bottle feeding. From the 5-minute recording, continuous waveforms of −10 mmHg or lower for 10 seconds or longer, without a pause exceeding 2 seconds, were selected for analysis (17) (Figure 1).

Intake volume was measured concurrently during the 5-minute sucking pressure assessment (17). Sucking pressures were measured during bottle feeding both with and without cheek support. Both measurements were obtained within a 12-hour interval to minimize the effect of maturation in each infant. Injections and eye examinations were avoided within 30 minutes prior to the measurement (17).

In previous studies, both jaw and cheek support were provided; however, this method makes it impossible for a single examiner to perform oral support and bottle holding simultaneously, and thus it is difficult to apply in clinical practice (11, 12). Therefore, in this study, we adopted a method commonly used by clinicians for premature infants, in which jaw support and bottle holding were performed with one hand.

**Figure 2:**
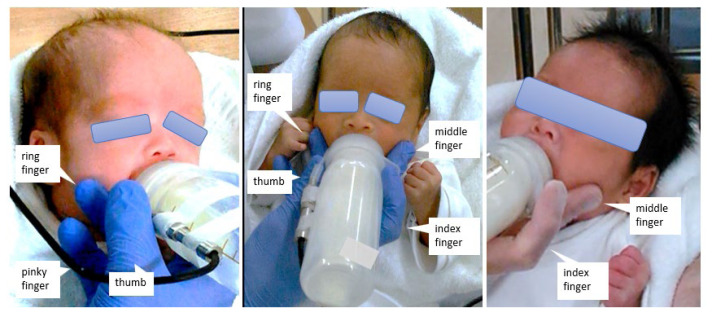
Cheek support for bottle feeding The bottle was held with thumb and index fingers, and middle and ring fingers compressed bilateral cheeks.

During bottle feeding, the investigator held the infant in a semi-upright supine position with the left hand. The method of cheek support for 5 minutes using the right hand is illustrated in Figure 2. The bottle was held with the thumb and index finger, while the bilateral cheeks were gently pressed using the middle and ring fingers. The strength of support was gentle so as not to harm the infant, and pressure was applied within a range that did not cause crying or signs of discomfort. If oxygen desaturation or bradycardia had occurred during sucking, the infant would have been allowed to rest (14, 17). Sucking pressures with and without cheek support were measured using the same type of milk under consistent respiratory conditions.

Cheek support during bottle feeding is illustrated in Figure 2. The bottle was held with the thumb and index finger, while the bilateral cheeks were gently pressed using the middle and ring fingers.

### Sucking pressure, number of burst and sucking for analysis and intake ratios

To evaluate sucking pressure, the mean sucking pressure (mmHg) was calculated, and the mean sucking pressure in bottle feeding with cheek support was divided by the mean sucking pressure in bottle feeding without cheek support to obtain the sucking pressure ratio (Figure 1). The number of bursts and sucking for analysis ratio was calculated by dividing the number of burst, sucking for analysis in cheek support by the number of burst, sucking for analysis in no support (Figure 1). The intake ratio was calculated by dividing the intake (mL) in cheek support by the intake (mL) in no support (Figure 1).

### Disclosure statement

The equipment used in the measurement of sucking pressure in this study (artificial nipple, silicone tubing, pressure transducer, amplifier, and data logger) was provided by Pigeon Co. Ltd. (Tokyo, Japan). The authors have no other conflicts of interest to disclose concerning this study.

## RESULTS

Four infants participated in this study. Their birth weight ranged from 612 to 2990 g, and their gestational ages at birth were between 24 and 37 weeks. On the day of measurement (with and without cheek support), postnatal age (PNA) ranged from 41 to 119 days, and modified weeks of postconceptional age (PCA) ranged from 39 to 51 weeks. Their weight ranged from 2216 to 4256 g (Table I).

The mean sucking pressures, number of burst and sucking, and intake volume during bottle feeding, both with and without cheek support, are summarized in Table II. In all cases, cheek support (pressing the cheeks) increased sucking pressure, the rest due to desaturation or bradycardia did not occur. The intake volume and the number of burst/sucking increased in two cases but decreased in the other two cases.

## DISCUSSION

Efficient sucking (1) is essential for establishing oral feeding and ultimately achieving hospital discharge. Oral support techniques, such as jaw and cheek support, have been reported to facilitate effective suckling in preterm infants (1, 10–12). However, no studies have examined the impact of oral support in non-preterm infants or assessed the individual effects of jaw and cheek support.

Therefore, in this study, we evaluated the effect of cheek support on sucking in four infants, including full-term cases.

Cheek support increased sucking pressure not only in preterm infants, but also in full-term infants with congenital diaphragmatic hernia and multicystic encephalomalacia. These results suggest that cheek support, even in the absence of jaw support, may improve sucking function.

A previous study (19) reported that sucking was influenced by body weight etc. Cases 1 and 4 which sucking pressure and intake increased, had even lower measurement weight less than Cases 2 and 3 which sucking pressure increased but intake did not. Therefore, since the lower body weight resulted in less suction, cheek support may have been more effective in Cases 1 and 4.

One possible mechanism underlying this improvement is that pressing the cheeks increases cheek stability and promotes lip seal (13). Another mechanism is that inward pressure on the cheeks compresses the buccal mucosa and narrows the intraoral cavity, thereby enhancing negative sucking pressure.

A previous study (18) reported that cheek stimulation (stroking the cheeks) enhanced the sucking rate. In contrast, in our study cheek support pressure was applied by a single examiner to keep consistency, the number of burst and sucking increased in two cases but decreased in the other two. This discrepancy may be attributed to differences in cheek pressing technique or the small sample size with varied comorbidities.

A previous study (11) had another area for future research was the examination of the parameters of sucking behavior in both intervention and nonintervention conditions, such as the number of sucks and the average amount of pressure. We showed we evaluated sucking pressure, number of burst/sucking through the measurement of the sucking pressure. Therefore, the method of measuring sucking pressure in this study showed the efficacy of analyzing cheek support.

This study has several limitations. We did not assess sucking pressure in relation to clinical significance. Thus, even if sucking pressure increases, it remains unclear whether feeding efficiency, weight gain, or earlier discharge are improved. A new study is needed that not only increases the number of cases for pressure measurement but also incorporates tracking of clinical outcomes.

Further studies with larger cohorts and broader clinical backgrounds are warranted to draw more definitive conclusions.

## CONCLUSION

In this study, we examined the effects of cheek support on oral feeding by measuring sucking pressure. Our findings suggested a possible association between cheek support and sucking pressure. Further studies with a larger number of cases will be required to determine whether cheek support can indeed enhance sucking pressure.

## Figures and Tables

**Table I:** Characteristics of infants at birth and at measurement date

		Birth		Measurement date				
	
		GA(weeks)	weight(g)	PNA(days)	PCA(weeks)	weight(g)	milk	Respiratory
1	ELBW	24	612	119	41	2216	breast	RA
2	CDH	37	2990	100	51	4256	breast	NC0.5L
3	LBW	33	1712	41	39	2570	formula	RA
4	LBW/ME	33	1508	41	39	2234	formula	RA

GA, gestational age; PNA, postnatal age; PCA, postconceptional age; ELBW, extremely low birth weight; LBW, low birth weight infant; CDH, congenital diaphragmatic hernia; ME, multicystic encephalomalacia; RA, room air; NC0.5L, nasal cannula at 0.5 L/min.

**Table II:** Characteristics of value of bottle feeding without and with cheek support

	Sucking pressure			Number of burst for analysis			Number of sucking for analysis			Intake		
			
	without*	with*	ratio	without	with	ratio	without	with	ratio	without	with	ratio
1	−54.46	−96.33	1.77	5	11	2.20	124	257	2.07	14	26	1.86
2	−134.39	−155.06	1.15	5	4	0.80	413	418	1.01	46	44	0.96
3	−245.68	−252.14	1.03	7	8	1.14	191	162	0.85	42	39	0.93
4	−166.84	−251.06	1.50	6	5	0.83	109	91	0.83	36	39	1.08

Sucking pressure values are expressed in mmHg. Amount of intake is expressed in mL.

* Values are expressed as means.
